# Regulatory T cells and IL10 suppress pulmonary host defense during early-life exposure to radical containing combustion derived ultrafine particulate matter

**DOI:** 10.1186/s12931-016-0487-4

**Published:** 2017-01-13

**Authors:** Sridhar Jaligama, Jordy Saravia, Dahui You, Nikki Yadav, Greg I. Lee, Bishwas Shrestha, Stephania A. Cormier

**Affiliations:** 1 Children’s Foundation Research Institute, Le Bonheur Children’s Hospital, 50 N Dunlap Street, Memphis, TN 38103 USA; 2 Institute for Plastic Surgery, School of Medicine, Southern Illinois University, Springfield, IL 62702 USA; 3 Department of Pediatrics, The University of Tennessee Health Science Center, Translational Research Science Bldg. 71S Manassas, Suite 430J, Memphis, TN 38103 USA

**Keywords:** Air Pollution, Particulate matter, EPFR, Influenza, Immunosuppression, Neonate, Treg, IL10

## Abstract

**Background:**

Exposure to elevated levels of particulate matter (PM) is associated with increased risk of morbidity and mortality due to respiratory tract viral infections in infants. Recent identification of environmentally persistent free radicals (EPFRs) in the PM from a variety of combustion sources suggests its role in the enhancement of disease severity of lower respiratory tract infections (LRTI). Our previous studies demonstrated that acute exposure to EPFRs induces pulmonary immunosuppression allowing for enhanced influenza disease severity. Here, we determine the mechanism of EPFR-induced immunosuppression and its impact on the immune response towards influenza infection.

**Methods:**

Neonatal mice (3 days old) were acutely exposed to DCB (combustion derived PM with chemisorbed EPFR) for seven consecutive days. Four days post-exposure (dpe), mice were infected with influenza virus. Pulmonary T cell phenotypes including regulatory T cells (Tregs) were analyzed by flow cytometry. The role of IL10 in EPFR-induced exacerbation of influenza disease severity was determined by administering recombinant IL10 (rIL10) to wild type mice or by using IL10 deficient (IL10^−/−^) neonatal mice. Mice were assessed for morbidity by measuring percent weight change and pulmonary viral load.

**Results:**

Neonatal mice exposed to EPFRs had a significant increase in pulmonary Tregs and the immunosuppressive cytokine IL10 following influenza infection, which coincided with decreased protective T cell responses to influenza infection at 6 dpi. Depletion of Tregs in EPFR-exposed neonatal mice resulted in increased protective, adaptive T cell responses, whereas adoptive transfer of Tregs from EPFR-exposed neonates to air-exposed neonatal mice suppressed adaptive T cell responses towards influenza infection. Further, treatment with rIL10 could recapitulate EPFR-induced exacerbation of morbidity and pulmonary viral load compared to air exposed and influenza infected mice, whereas, EPFR-exposed IL10^−/−^ neonates exhibited significant reductions in morbidity, pulmonary viral load and adaptive T cell responses following influenza infection.

**Conclusions:**

Neonatal exposure to EPFRs induced Tregs and IL10 resulting in suppressed adaptive T cell responses and enhanced influenza disease severity in neonatal mice. Depletion of Tregs increased adaptive T cell responses and deficiency of IL10 reduced morbidity and conferred enhanced protection against influenza virus.

**Electronic supplementary material:**

The online version of this article (doi:10.1186/s12931-016-0487-4) contains supplementary material, which is available to authorized users.

## Background

Exposure to elevated levels of airborne particulate matter (PM) pollution has been associated with adverse health outcomes such as asthma and chronic obstructive pulmonary disease (COPD) [1, 2]. Interestingly, several epidemiological studies have also recognized an association between exposure to elevated levels of PM and respiratory tract viral infections in infants and young children [3–5]. Due to the heterogeneous nature of PM, the associated health effects of exposure to elevated levels of PM could not be attributed to a specific component. Recent studies by our collaborators identified the presence of “environmentally persistent free radicals” (EPFRs) in PM samples that were collected in various cities of United States [6]. Though epidemiological studies suggest that infants and young children are susceptible to adverse health effects of PM exposure [7], very limited mechanistic information is available to understand the impact of PM exposure in determining the outcome of respiratory tract infections in this immunologically immature subpopulation. Our previous studies demonstrated that acute early-life exposure to EPFR containing PM induces oxidative stress, inflammation and airway hyper-responsiveness in the lungs [8]. These responses were accompanied by airway epithelial barrier dysfunction and pulmonary remodeling as evidenced by airway smooth muscle thickening as a result of epithelial to mesenchymal transition [9]. We demonstrated that exposure to EPFRs induces a temporary immunosuppressive pulmonary environment in an oxidative stress dependent manner resulting in enhanced disease severity following influenza virus infection [10, 11]. Specifically, we observed reduced adaptive T cell responses including reductions in protective T cell responses (i.e. Tc1 and Th1) following influenza infection [10]. While we expected reductions in protective T cell responses due to the age of infection [12], EPFR exposure further exacerbated disease as was evidenced by further reductions in protective T cell numbers and enhanced influenza morbidity and mortality.

In the current study, we sought to determine the mechanism of pulmonary immunosuppression observed during early-life exposure to EPFRs and its role in the exacerbation of the influenza disease severity. Since we had previously observed an increase in Tregs in the lungs of neonatal mice exposed to EPFRs [11], we hypothesized that Tregs, and their effector cytokine IL10, were critical for EPFR-induced exacerbation of influenza disease severity in neonates. To address this hypothesis, we exposed neonatal mice deficient in Tregs to EPFRs and then infected them with influenza virus. Corollary experiments in which Tregs from EPFR-exposed neonatal mice were adoptively transferred into air-exposed neonatal mice were then performed to demonstrate the direct role of EPFR-induced Tregs in mediating the suppression of adaptive T cell responses and exacerbation of influenza disease severity. We also studied whether IL10 was the effector molecule responsible for EPFR-enhanced flu severity in neonatal mice. Our data demonstrates a mechanism whereby exposure to EPFRs alter pulmonary immune responses via the induction of Tregs and IL10 and modulate the protective immune responses against influenza infection.

## Methods

### Combustion derived PM

Radical containing combustion derived ultrafine PM with a mean aerodynamic diameter of ~0.2 μm was generated and characterized by our colleague Dr. Slawo Lomnicki at Louisiana State University as described earlier [8, 13]. Briefly, particles are composed of 95% silica and 5% CuO and vapors of 1,2-dichlorobenzene are dosed as an adsorbate inside a custom made vacuum exposure system for 5 min at 10 torr at 230 °C. The surface area of these particles is 460 m^2^/g and the density is 2.2 g/cm^3^. Our previous studies determined the size of the particles in a monodispersion and the mean aerodynamic diameter (MAD) of these particles was found to be 0.195 μm. Analysis using flow cytometry and transmission electron microscopy (TEM) showed that particles once suspended were monodispersed with little or no aggregates [14]. Further, our previous studies demonstrated that aerosol delivery of ultrafine particles under a constant rate could deposit the particles in the lower respiratory tract of neonatal rodents [15]. For aerosol delivery, DCB particles (PM with chemisorbed EPFRs previously published as DCB230 [10]) were suspended in 25 ml of irrigation saline containing 0.02% Tween-80 at a final concentration of 0.2 mg/ml. Particles were monodispersed by probe sonication immediately prior to exposure.

### Animals

C57BL/6NHsd breeders were originally purchased from Harlan (Indianapolis, IN). IL10 knock out mice (B6.129P2-I*l*10^tm1Cgn^/J) (stock number-002251) were purchased from Jackson laboratories (Bar Harbor, ME). These mice were homozygous for the *Il10* ^*tm1Cgn*^ targeted mutation. Neonatal mice were not identified for sex and both sexes were used for experiments. Separate cohorts of mice were used for independent experimental endpoints. All mice were housed in ventilated cages and supplied with filtered air in a specific pathogen free environment with free access to food and water under controlled conditions with 12 h light/dark cycle, temperature and humidity. Mice were time-mated to obtain maximum number of birth cohorts. All animal protocols were prepared and followed in accordance with Guide for the Care and Use of Laboratory Animals and were approved by the Institutional Animal Care and Use Committee.

### Neonatal exposure to EPFRs and influenza infection

Three day old neonatal mice were exposed to DCB at 200 μg/m^3^ or filtered air for 30 min/day for seven consecutive days. Modeling data (MPPD v2.0) was used to produce an equivalent particle deposition per alveolus compared to that of an infant human being exposed over a 24 h time period to an average of 35 μg/m^3^ of PM_2.5_. The physiological parameters that were incorporated in the algorithm and the calculations used to determine air flow in the chamber were described previously [11]. Recent development of computational methods to quantify the transport and deposition of particles in human respiratory tract suggests the deposition of ultrafine particles in the lower respiratory tract [16]. Since respiratory mechanical parameters in mice were found to be similar to rats [17], mouse modeling data was obtained from literature and default rat lung parameters were overwritten to correct for physiological parameters with the assistance of Drs. Price and Asgharian (MPPD support). At four days post exposure (dpe), neonates were infected intranasally (i.n.) with mouse-adapted human influenza A strain (PR/8/34 H1N1) (Advanced Biotechnologies, MD) at 1.25 TCID_50_/neonate diluted in 10 μl Dulbecco’s Phosphate-Buffered Saline (DPBS) or sham infected with the same volume of DPBS.

### Pulmonary viral load

Neonatal mice were sacrificed at 4 days post-infection (dpi) and lungs were isolated. Lungs were homogenized and peak pulmonary viral loads were determined by using Reed-Muench calculator [18]. Briefly, Madin-Darby Canine Kidney (MDCK) epithelial cells were seeded at a density of 1.2× 10^4^ cells/well in a 96 well plate. Lungs isolated at 4dpi were homogenized in 1 ml DPBS on ice and centrifuged at 2000xg for 10 min at 4 °C. Cells were inoculated with a 10-fold dilution series of the supernatant obtained from the homogenized lungs and incubated at 37 °C with 5% CO_2_ for five days. Signs of influenza-induced cytopathic effect was determined daily and the scoring for cytopathic effect was finalized on day five and TCID_50_ was calculated.

### Depletion of Tregs

Tregs were depleted in the lungs of neonatal mice using the purified anti-mouse CD25 antibody (PC61 clone) (BioLegend, San Diego, CA). Briefly, 5 μg of anti-CD25 antibody in 10 μl volume was administered i.n. to neonatal mice every two days on days 0 and 3, and 6 dpe at 2 h prior to the exposure to DCB. Control mice received similar amount of rat IgG isotype control antibody (10 ﻿μl). All mice were exposed to DCB from day 3 to day 9 of age for seven consecutive days. Treg depletion efficiency was determined at 4 dpe to verify whether Tregs were depleted before infection and also at 10 dpe to verify whether depletion is maintained until 10 dpe. Lungs were isolated on days 4 dpe and 10 dpe and a single cell suspension was prepared to stain for markers CD3, CD4, CD25, and transcription factor FoxP3. Percentage of Tregs in the lung was determined using flow cytometry and cells positively stained for surface markers CD3, CD4, CD25 and transcription factor Foxp3 were considered Tregs.

### Treg Adoptive Transfer

Lungs were isolated from DCB exposed neonatal mice at 10 dpe. Single cell suspension of the isolated lungs was pre-enriched for CD4^+^ T cells using biotinylated anti-mouse CD4 antibody and Cellection Biotin Binder kit (Life Technologies, Grand Island, NY), following manufacturer’s instruction. Subsequently, CD4^+^ enriched fraction was used to isolate CD4^+^CD25^+^ cells (Tregs) using Easysep Mouse CD4^+^CD25^+^ Regulatory T cell isolation kit (Stem cell Technologies, Vancouver, Canada), following manufacturer’s instructions. The amount of Tregs transferred into each mouse was equivalent to the amount we observed in the lungs of DCB treated mice plus 30% extra to account for losses during transfer. Thus, since we observed ~5.0*10e5 CD25^+^ Tregs in the lungs of EPFR exposed neonatal mice, we transferred ~6.4*10e5 CD4^+^CD25^+^ Tregs in 10 μl of serum free media to air-exposed neonatal mice i.n. 2 h prior to infection with influenza virus. All mice were sacrificed on 6 dpi (10 dpe) and analyzed for Th1 and Tc1 responses by means of intracellular cytokine staining and flow cytometry.

### Treatment with recombinant IL10 (rIL10)

To understand the effect IL10 in EPFR-induced exacerbation of influenza disease severity, air exposed WT neonatal mice were treated with rIL10 (BioLegend, San Diego, CA) (150 pg per mice via i.n. route) at 4 dpe. Mice were subsequently infected with influenza virus (1.25 TCID_50_per mouse) at 2 h-post transfer. The amount of IL10 administered was equivalent to IL10 observed in EPFR-exposed mice at 4dpe and total amount instilled was adjusted for transfer efficiency (i.e. +30%).

### Flow cytometry and intracellular staining

Mice were sacrificed and blood was gently cleared from the lungs using retrograde vascular perfusion using Hank’s Balanced Salt Solution (HBSS). Lungs were isolated and a single cell suspension was prepared as described earlier [10, 19]. Briefly, lungs were dissected and collected into 2.4 ml ice-cold HBSS containing 1 mg/ml collagenase I (Invitrogen NY) and 150 ng/ml DNase (Sigma Aldrich, NY) per neonatal lung and mechanically dissociated using Octodissociator (Miltenyi, Germany). Dissociated lungs were incubated at 37 °C for 30 min with continuous shaking (200 rpm). Following incubation, lungs were dissociated with Octodissociator for a second time and remaining cell clumps were filtered through 40 μm cell strainer to obtain a single cell suspension. The resulting cell suspension was treated with RBC lysis buffer (BioLegend, San Diego, CA) to lyse residual RBCs. For intracellular staining, cells were stimulated for five hours at 37 °C in RPMI 1640 media containing 5% heat-inactivated fetal bovine serum, 5 ng/ml phorbol 12-myristate 13-acetate, 500 ng/ml Ionomycin (Sigma-Aldrich) in the presence of a protein transport inhibitor (GolgiPlug, BD Biosciences, San Jose, CA). T helper-1 cells (Th1) subsets were identified as CD3^+^, CD4^+^, and IFNγ^+^ and cytotoxic T cell-1 (Tc1) subsets were identified as CD3^+^, CD8^+^, and IFNγ^+^. Tregs were identified as CD3^+^, CD4^+^ CD25^+^, and intracellular Foxp3^+^ cells. The antibodies used for intracellular staining were: eFluor450-CD3 (clone- 17A2; eBioscience, CA), FITC- CD8 (clone- 53–6.7; eBioscience), PerCP-CD4 (clone- RM4-5; BioLegend, CA), and PE-IFNγ (clone- XMG1.2; eBioscience). For Treg staining, following antibodies were used eFluor450-CD3 (clone-17A2; eBioscience), PerCP-CD4 (clone - RM4-5; BioLegend), PE-CD25 (clone- PC61.5; eBioscience), and FITC-Foxp3 (clone- FJK-16 s; eBioscience). Fixable live/dead marker eFluor 780 (eBioscience, San Diego, CA) was used to exclude dead cells from analysis. Following antibody staining, cells were analyzed with FACS Canto II (BD Biosciences) and flow cytometry data was analyzed using FlowJo Software v.10.1 (Tree Star, OR).

### IL10 and TNFα ELISA

Lungs were isolated from neonatal mice following retrograde vascular perfusion. Isolated lungs were flash frozen in liquid nitrogen and stored at -80 ^o^C until processed further for cytokine analysis. Briefly, lungs were homogenized in 1 ml of Tissue Protein Extract Reagent (T-PER) (Life Technologies, Grand Island, NY) containing protease inhibitor cocktail (Thermo Scientific, Waltham, MA). Homogenates were centrifuged at 10,000xg for 5 min and supernatants were collected for analysis. IL10 levels were determined by mouse IL10 quantikine ELISA kit following manufacturer’s (R&D systems, Minneapolis, MN) instructions. TNFα levels were measured using Legend Max mouse TNFα ELISA kit (BioLegend, San Diego, CA) following manufacturer’s instructions. Data was normalized to the total protein content in the supernatants as determined by BCA protein assay (Life Technologies, Grand Island, NY).

### Immunohistochemistry

Neonatal mice were sacrificed and lungs were excised and inflated at a constant fluid pressure of 25 cm with Zinc-formalin and fixed overnight as described earlier [11]. Subsequently, the lungs were transferred to 70% ethanol until ready for embedding. Lungs were dehydrated, paraffin-embedded and sectioned into 5 μm thick sections and mounted onto glass slides. Immunohistochemical staining was performed incubating with rabbit polyclonal anti-human H1N1 influenza virus antibody (Clontech Laboratories, Mountain View, CA) at a concentration 5 μg/ml in PBS containing 1% BSA. Tissue sections were washed with Tris Buffered Saline containing 0.5% Tween 20 (TBST) and incubated with anti-rabbit IgG (ImmPRESS anti-rabbit IgG kit, Vector Laboratories, Burlingame, CA). Following incubation, sections were washed in TBST and incubated with peroxidase substrate solution until desired stain intensity was developed. Sections were further counter stained with hematoxylin and observed for lung areas positively stained for influenza virus.

### Statistics

All results were expressed as mean ± SEM and were analyzed using GraphPad Prism 6 (GraphPad Software Inc., Version 6.0.7, La Jolla, CA). One-way ANOVA or Two-way ANOVA with Tukey’s multiple comparison’s test with Holm-Sidak’s correction for multiple comparisons, and unpaired t-tests were performed to determine the difference between groups. p values less than 0.05 were considered statistically significant.

## Results

### Treg and IL10 are induced after exposure to EPFRs and influenza infection

**Fig. 1:**
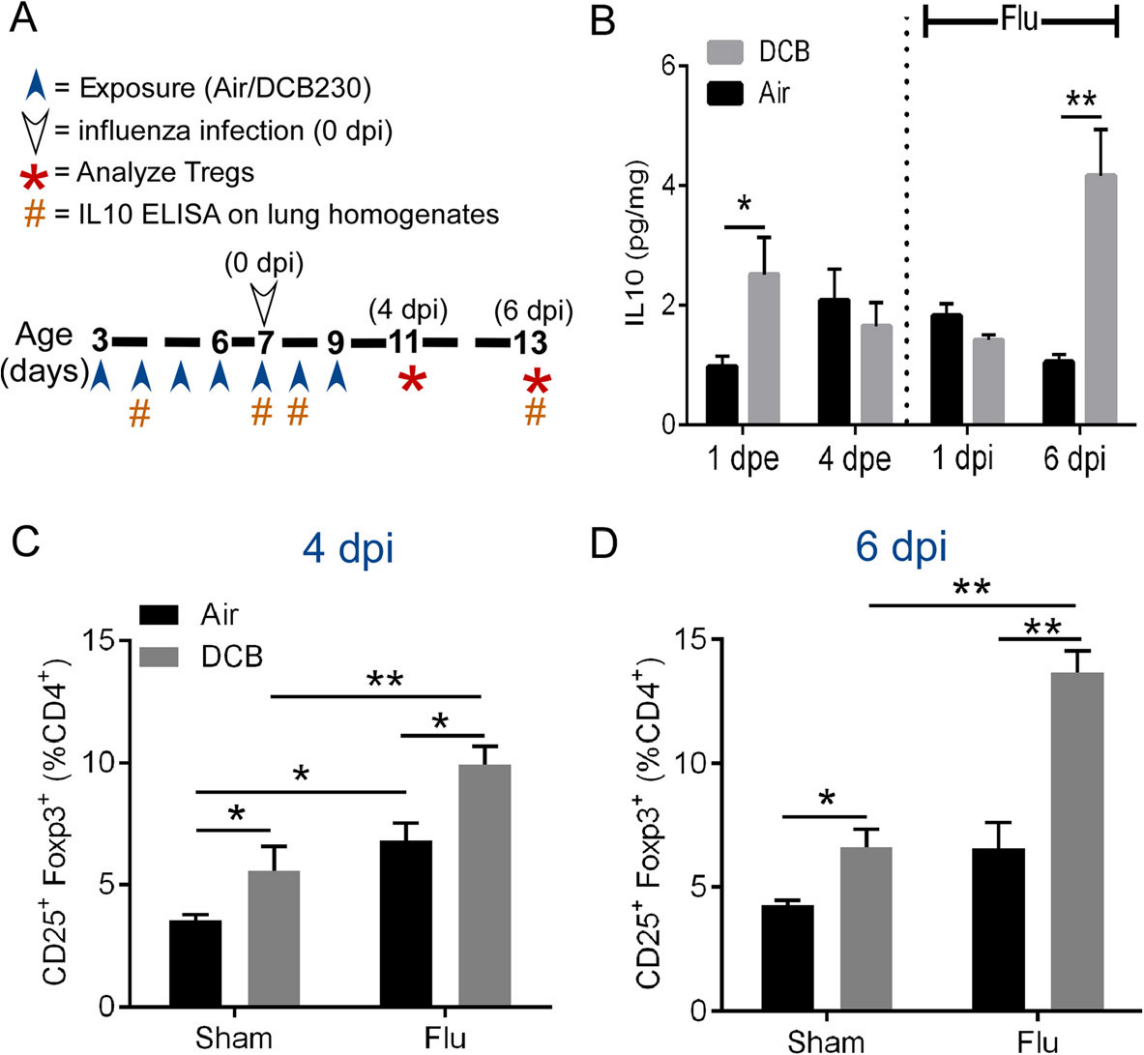
Effect of EPFR exposure on pulmonary Treg induction and IL10 induction. **a** Timeline demonstrating the exposure of 3 day old neonatal mice to Air or DCB particles (200 μg/m^3^) for seven consecutive days (*blue arrow heads*) from day 3 to day 9 of age (0–6 dpe) for 30 min/day. Mice were infected with mouse-adapted influenza A virus (PR/8/34 strain) (white arrow head) at 1.25 TCID_50_ on 4dpe (0 dpi). At 4 and 6 dpi, lungs were isolated from mice exposed to Air or DCB and infected i.n. with influenza (Air/Flu and DCB/Flu) or sham infected with DPBS (Air/Sham and DCB/Sham). Single cell suspensions were prepared to identify and quantify pulmonary Tregs by flow cytometry. **b** Time-course of IL10 levels after exposure to EPFRs and influenza infection. Lungs were isolated on 1 and 4 dpe and on 1 and 6 dpi and whole lung homogenates were analyzed for IL10 levels. **c**, **d** Treg (percentage CD4^+^ cells expressing CD25 and Foxp3) frequency in Air/Sham, DCB/Sham, Air/Flu, and DCB/Flu at 4 and 6 dpi, respectively. Data were plotted as mean ± SEM. *p < 0.05 and ** *p* < 0.01. **b** multiple *t*- tests with Holm-Sidak correction for multiple comparisons and (**c**, **d**), Two-way ANOVA with Tukey’s multiple comparison test

Our previous studies demonstrated that acute inhalation exposure to combustion derived PM induced immunosuppression as evidenced by increase in Tregs and increased expression of IL10 in CD4^+^ T cells and dendritic cells [10, 11]. However, these studies failed to demonstrate a causal role for Treg and/or IL10 in influenza severity. To address this question, we first examined the kinetics of Treg and IL10 induction during the exposure to Air/EPFRs during influenza infection (Air/Flu or DCB/Flu). Analysis of IL10 levels in whole lung homogenates over the course of the exposure to EPFRs revealed an early (1 dpe) increase that returned to baseline levels by 4 dpe (prior to influenza infection) and remained at baseline unless mice were infected with influenza (data not shown). Upon infection with influenza, we observed a second induction of IL10 (6 dpi in DCB/Flu lungs) (Fig. 1b). The later induction of IL10 coincided with our previously reported suppression of adaptive T cell responses [11] at 6 dpi. ﻿TNFα levels were found to be below the ﻿limits ﻿of detection (1.5 pg/ml) in Air and DCB exposed lungs at 1 and 4 dpe and in Air/Flu and DCB/Flu mice at 1 dpi. Only 3 of the DCB/Flu mice (n = 6) had detectable levels of TNFα at 6 dpi (Additional file 1). Tregs (CD4^+^, CD25^+^, and Foxp3^+^) cells were quantified following EPFR exposure and influenza infection at 4 and 6 dpi using flow cytometry. The percentage Tregs were significantly increased in DCB exposed mice compared to Air exposed mice at both 4 and 6 dpi. Furthermore, Tregs were significantly increased in DCB/Flu mice compared to Air/Flu mice at both 4 and 6 dpi (Figs. 1c and 1d ). The percent of Tregs in influenza infected Air (Air/Flu) or DCB exposed (DCB/Flu) mice were significantly higher compared to Air or DCB exposed and sham infected mice, respectively at 4 dpi (Fig. 1c). The percent of Tregs in DCB/Flu mice were also significantly higher than DCB exposed and sham infected mice at 6 dpi, (Fig. 1d).

### Depletion of Tregs in EPFR exposed mice restores adaptive T cell response against influenza

**Fig. 2:**
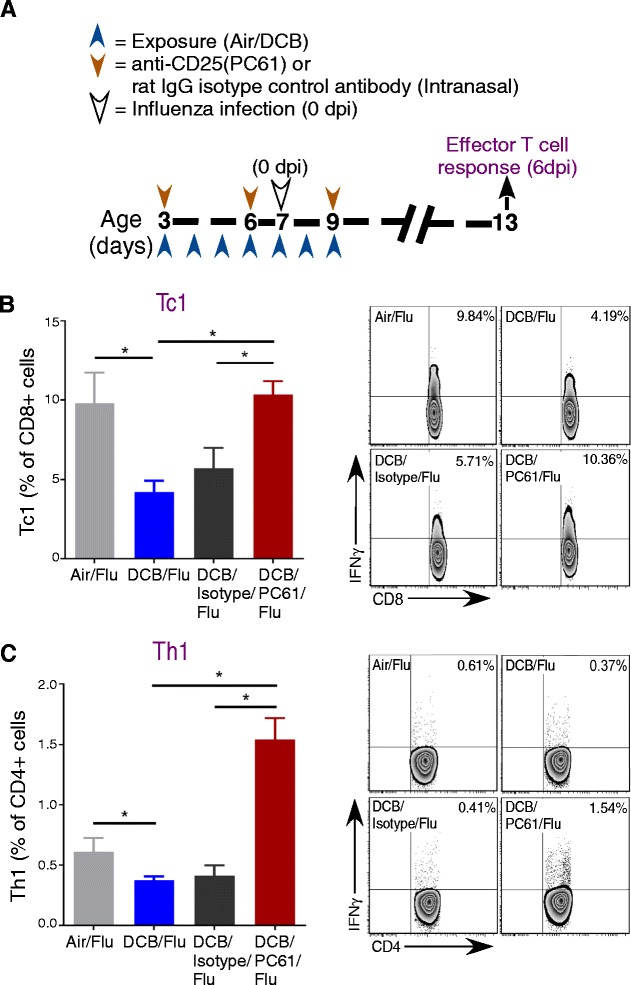
Depletion of Tregs in the lungs of DCB-exposed neonatal mice enhances adaptive T cell responses. **a** Schematic for DCB exposure, influenza infection, administration of anti-CD25 (PC61 clone) monoclonal antibody or rat IgG isotype control, and the analysis of adaptive T cell responses at 6 dpi. **b** Percentage of CD8^+^ cells expressing IFNγ (Tc1) and representative flow contour plots in Air/Flu, DCB/Flu, DCB/Isotype/Flu, and DCB/PC61/Flu mice. **c** Percentage of CD4^+^ cells expressing IFNγ (Th1) and representative flow contour plots in Air/Flu, DCB/Flu, DCB/Isotype/Flu, and DCB/PC61/Flu mice. *p < 0.05. Data were plotted as mean ± SEM. One-way ANOVA with Tukey’s multiple comparison test

Since our previous studies demonstrated Treg induction and suppression of adaptive T cell responses (decreased CD8^+^ IFNγ^+^ (Tc1) and CD4^+^ IFNγ^+^ (Th1) cells numbers) after exposure to EPFRs [10], we studied whether depletion of Tregs would restore the adaptive T cell response towards influenza. Neonatal mice were exposed to EPFRs as described earlier. To deplete Tregs, we administered 5 μg of anti-CD25 (PC61) antibody every 2 days (i.n.) to a subset of mice. The efficiency of depletion was confirmed at both 4 dpe and 10 dpe (Additional file 2A) compared to DCB exposed and rat IgG isotype control antibody treated neonatal mice (DCB/Isotype Ctrl) (Additional file 2B and C). To determine if Treg depletion increased protective, effector T cell responses, mice receiving isotype or PC61 antibody were exposed to air or DCB and infected with influenza (Fig. 2a). Lungs were isolated at 6 dpi and pulmonary T cell profiles were assessed by flow cytometry. The percent of both Tc1and Th1 cells were decreased in DCB/Flu mice compared to Air/Flu mice (Fig. 2b and 2c, respectively). However, DCB/PC61/Flu mice, which lacked Tregs, exhibited a significant increase in both Tc1 (Fig. 2b) and Th1 (Fig. 2c) cells compared to DCB/Flu or DCB/Isotype/Flu mice. A significant decrease in IL10 was observed in DCB/PC61/Flu mice at 6 dpi compared to DCB/Flu and DCB/Isotype/Flu mice. No significant difference in IL10 was observed between DCB/Flu and DCB/Isotype/Flu mice (Additional file 3).

### Adoptive transfer of Tregs from EPFR exposed mice suppresses pulmonary adaptive T cell responses in Air-exposed mice after influenza infection

**Fig. 3:**
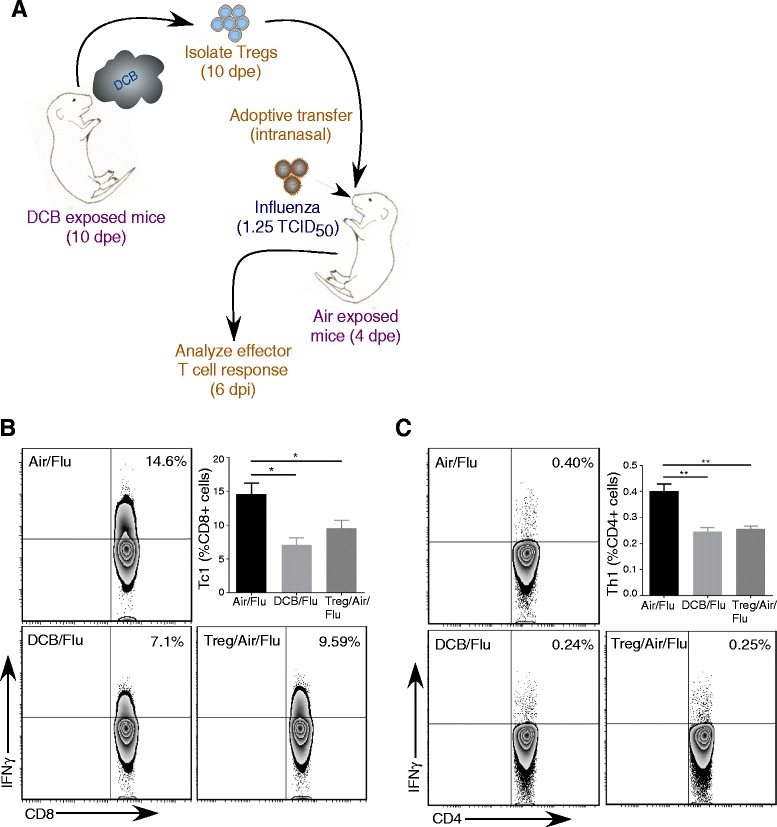
Tregs from EPFR-exposed mice suppresses adaptive T cell response in Air-exposed mice. **a** Schematic for isolation and adoptive transfer of Tregs from DCB exposed mice to Air exposed mice at 10 dpe, i.n. influenza infection and analysis of adaptive T cell response at 6 dpi. **b** Percentage of pulmonary CD8^+^ cells expressing IFNγ (Tc1) and representative flow contour plots in DCB/Flu, and Treg/Air/Flu mice in comparison to Air/Flu mice. **c** Percentage of pulmonary CD4^+^ cells expressing IFNγ (Th1) and representative flow contour plots in DCB/Flu, and Treg/Air/Flu mice in comparison to Air/Flu mice. Data are plotted as means ± SEM. *p < 0.05 and ** *p* < 0.01 vs Air/Flu mice. One-way ANOVA with Holm-Sidak correction for multiple comparisons

To further delineate the role of EPFR-induced Tregs in mediating the suppression of adaptive T cell responses and exacerbation of influenza disease severity, Tregs were isolated from DCB exposed mice at a time point during ﻿which﻿﻿ peak Treg responses were observed (6 dpi) and adoptively transferred into the lungs of Air- exposed mice at 4 dpe. The efficiency of isolation was confirmed by flow cytometry and the percentage CD25^+^ cells after isolation was found be approximately 90% (Additional file 4). Following transfer of Tregs, mice were infected with influenza virus at 1.25 TCID_50_ /mouse (Fig. 3a). At 6 dpi, lungs were isolated and Tc1 and Th1 responses were analyzed by flow cytometry. Air exposed mice that received Tregs from DCB exposed mice (Treg/Air/Flu) exhibited a significant decrease in Tc1 and Th1 cells compared to Air/Flu mice (Fig. 3b and 3c, respectively). Further, the level of Tc1 and Th1 cell suppression in Treg/Air/Flu mice was found to be similar to DCB/Flu mice.

### IL10 recapitulates EPFR-induced exacerbation of influenza morbidity

**Fig. 4:**
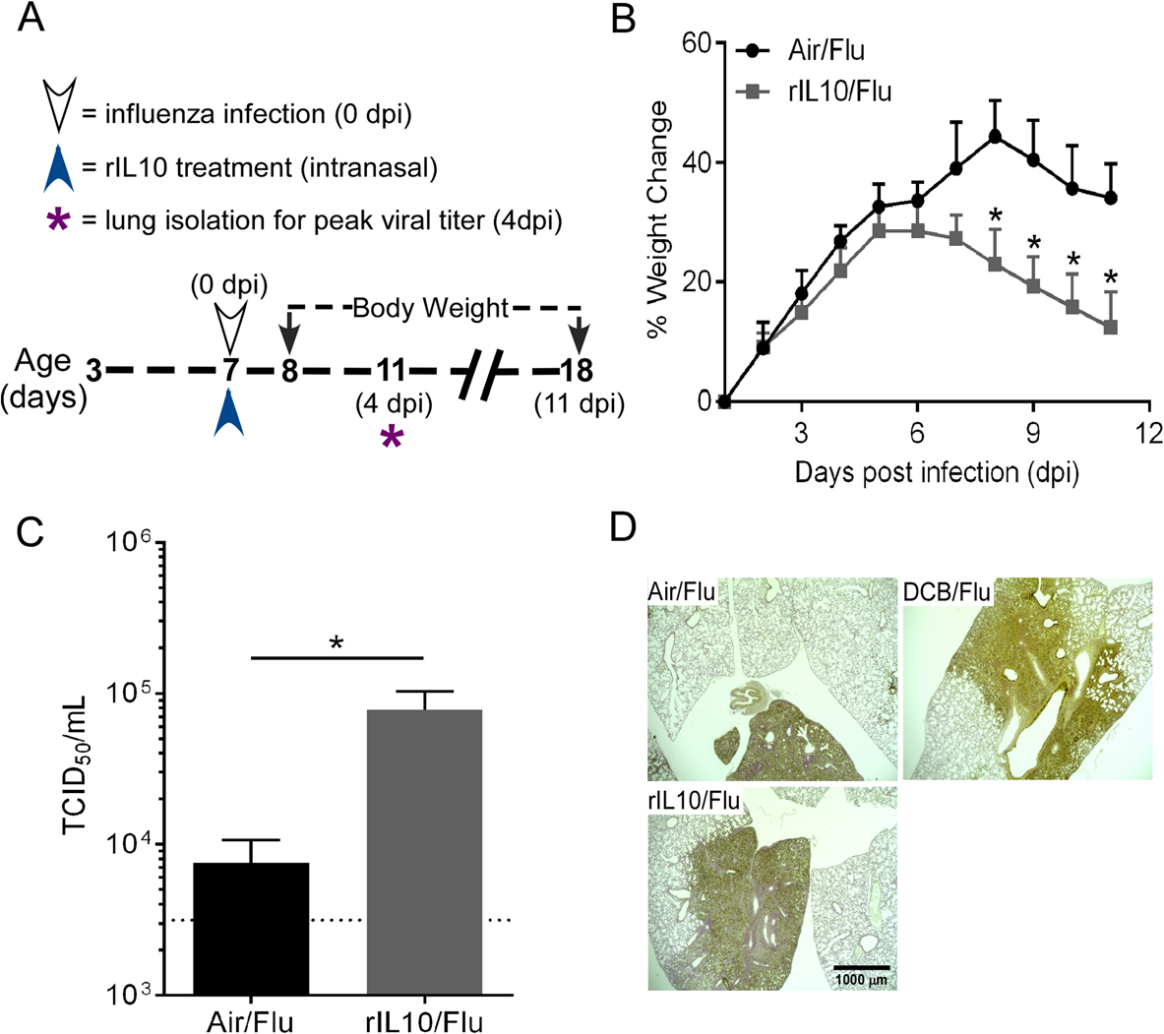
IL10 recapitulates the EPFR-induced exacerbation of influenza morbidity in Air-exposed mice. **a** Schematic for rIL10 administration and i.n. influenza infection and assessment of percentage weight change and peak pulmonary viral load at four dpi. **b** Average percent body weight change ((value-baseline/baseline)*100) over a period of 11 dpi in Air-exposed mice treated with rIL10 and subsequently (2 h-post treatment) infected with influenza virus. **c** Pulmonary viral load in Air/Flu and rIL10/Flu mice at 4 dpi. Limit of detection is indicated by the dotted line. **d** Immunohistochemical staining for influenza virus in the lungs of Air/Flu, DCB/Flu, and rIL10/Flu mice at 4 dpi. Data were plotted as means ± SEM; *p < 0.05 and **p < 0.01; (**b**) Multiple comparisons t tests with Holm-Sidak correction for multiple comparisons and (**c**) Student’s *t*-test

Our previous studies demonstrated that acute exposure to EPFRs induces an immunosuppressive environment that was associated with increased levels of IL10 in the lungs of EPFR exposed neonatal mice [11]. However, these studies did not report the role of EPFR-induced IL10 in mediating the increase in severity of influenza disease. Since we observed an initial increase in IL10 at 1 dpe which peaked at 6 dpi, we studied whether external rIL10, if administered to Air-exposed neonatal mice would recapitulate the EPFR induced exacerbation of influenza disease severity. Body weight gain was assessed as a measure of influenza disease associated morbidity and peak pulmonary viral load was assessed at 4 dpi as a measure of influenza disease severity. We administered rIL10 (i.n.) to neonatal mice at 7 days of age (age match for 4 dpe) and infected them with influenza virus (1.25 TCID_50_) 2 h post-transfer (Fig. 4a). rIL10/Air/Flu mice once infected exhibited greater reductions in weight gain (i.e. reduced percent weight change) compared to Air/Flu mice (Fig. 4b). Further, rIL10/Air/Flu mice exhibited significantly higher pulmonary viral loads at 4 dpi compared to Air/Flu mice (Fig. 4c). Body weight change and pulmonary viral load in rIL10/Flu mice were found to be similar compared to our previous observation in EPFR exposed influenza infected neonatal mice [10]. Consistent with these observations, immunohistochemistry for influenza virus on rIL10/Flu lungs demonstrated significantly larger infected areas compared to Air/Flu lungs (Fig. 4d). Together, these observations demonstrate that administration of rIL10 recapitulates the effect of EPFRs in exacerbating influenza disease severity in neonatal mice.

### Effect of IL10 deficiency on the morbidity, pulmonary viral load and adaptive T cell response following EPFR exposure and influenza infection in neonatal mice

**Fig. 5:**
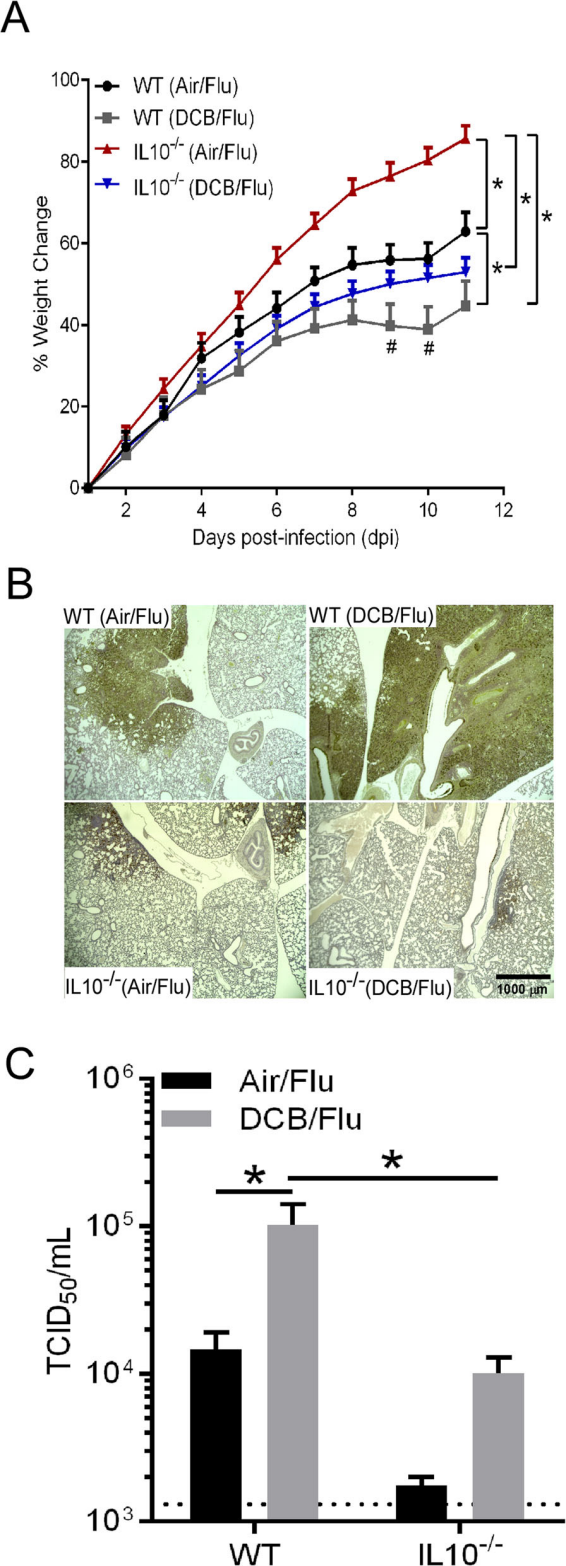
Effect of IL10 deficiency on the morbidity and pulmonary viral load in EPFR-exposed neonatal mice. **a** Average percent body weight change ((value-baseline/baseline)*100) in WT and IL10^−/−^ mice exposed to Air or DCB. **b** Immunohistochemical staining for influenza virus representing the pulmonary viral load at 4 dpi in WT and IL10^−/−^ mice exposed to Air or DCB. **c** Pulmonary viral load at 4 dpi in WT and IL10^−/−^ mice exposed to Air or DCB and infected with influenza virus. Limit of detection is indicated by dotted line. Data were plotted as means ± SEM; *p < 0.05, WT (Air/Flu) vs WT (DCB/Flu), IL10^−/−^ (Air/Flu) vs IL10^−/−^ (DCB/Flu), WT (Air/Flu) vs IL10^−/−^ (Air/Flu), and IL10^−/−^ (Air/Flu) vs WT (DCB/Flu); ^#^ *p* < 0.05, WT (DCB/Flu) vs IL10^−/−^ (DCB/Flu) on indicated dpi. **a** Multiple comparisons t tests with Holm-Sidak correction for multiple comparisons (**c**) Two-way ANOVA with Holm-Sidak correction for multiple comparisons test

**Fig. 6:**
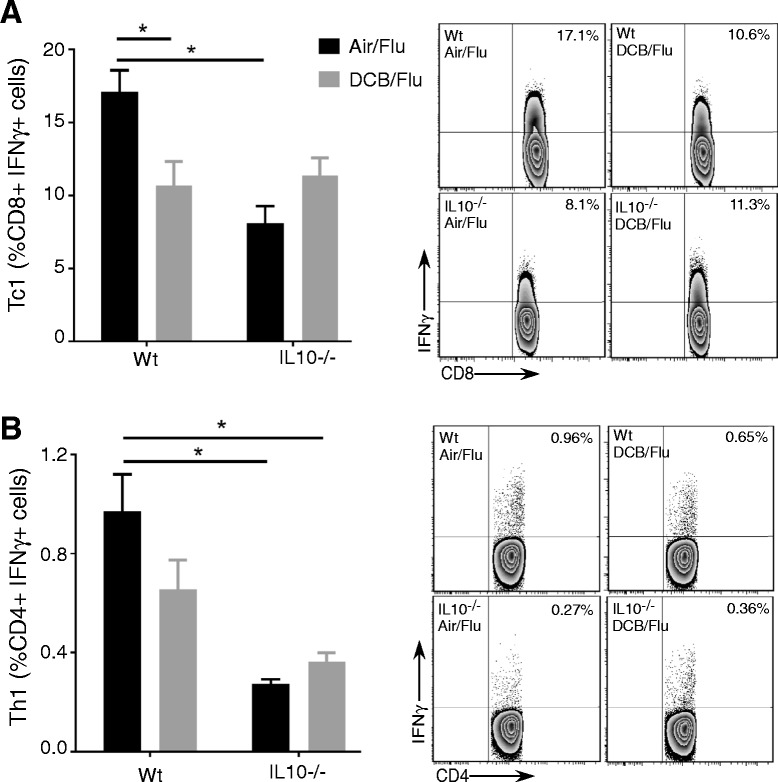
Effect of IL10 deficiency on adaptive T cell response in EPFR exposed and influenza infected neonatal mice. **a** Percentage of pulmonary CD8^+^ cells expressing IFNγ (Tc1) and representative flow contour plots in WT and IL10^−/−^mice after exposure to Air or DCB and influenza infection at 6 dpi. **b** Percentage of pulmonary CD4^+^ cells expressing IFNγ (Tc1) and representative flow contour plots in WT and IL10^−/−^mice after exposure to Air or DCB and influenza infection at 6 dpi. Data are plotted as means ± SEM, *p < 0.05. Two-way ANOVA with Holm-Sidak’s multiple comparisons test

To further understand the role of IL10 in mediating EPFR-induced immunosuppression and further leading to exacerbated influenza disease severity, we exposed mice deficient in IL10 (B6.129P2-Il10^tm1Cgn^/J; IL10^−/−^) to EPFRs and infected them with influenza. Neonatal wildtype (WT) and IL10^−/−^ mice were exposed to Air or EPFRs as described earlier and infected with influenza virus on 4 dpe. Body weight gain, peak pulmonary viral load (4dpi), and adaptive T cell responses (6dpi) were analyzed. A significant decrease in the percent body weight change was observed in DCB exposed WT mice (blue line) compared to the Air exposed WT mice (black line). A significant decrease in the percent body weight change was observed in DCB exposed IL10^−/−^ mice (IL10^−/−^(DCB/Flu); grey line) compared to Air exposed IL10^−/−^ mice (IL10^−/−^Air/Flu; red line). However, the percent body weight change was slightly greater in IL10^−/−^(DCB/Flu) mice and was statistically different at 9 and 10 dpi compared to WT(DCB/Flu) suggesting that IL10 deficiency can at least partially reduce the EPFR-induced exacerbation of influenza-associated morbidity. Further, the percent body weight change in IL10^−/−^(Air/Flu) mice was significantly higher compared to WT(Air/Flu) mice suggesting that IL10^−/−^ mice are less susceptible to influenza associated morbidity (Fig. 5a). Consistent with these observations, a significant increase in pulmonary viral load was observed at 4 dpi in WT(DCB/Flu) mice compared to WT(Air/Flu) mice (Fig. 5c), whereas, smaller areas of the lungs were infected with influenza (Fig. 5b) as evidenced by immunohistochemistry for influenza virus and a significant decrease in peak pulmonary viral load at 4 dpi (Fig. 5c) was observed in IL10^−/−^(DCB/Flu) mice compared to WT(DCB/Flu) mice. It is interesting to note that the loss of IL10 resulted in lower viral loads even in the WT(Air/Flu) mice; there was no significant difference between viral loads in the IL10^−/−^ vs. WT mice. To study the effect of IL10 deficiency, on adaptive T cell responses against influenza virus following EPFR exposure, lungs were isolated on 6 dpi and T cell profiles were assessed. The percentage Tc1 and Th1 cells were significantly lower in WT(DCB/Flu) neonates compared to WT(Air/Flu) neonates (Figs. 6a and b, respectively). However, the percentage of Tc1 and Th1 cells were significantly lower in IL10^−/−^(Air/Flu) and IL10^−/−^(DCB/Flu) neonates compared to WT/Air/Flu neonates (Figs. 6a and b, respectively). Further, the percent Tc1 cells observed in IL10^−/−^(DCB/Flu) mice was significantly greater compared to IL10^−/−^(Air/Flu) mice (Fig. 6a).

**Fig. 7:**
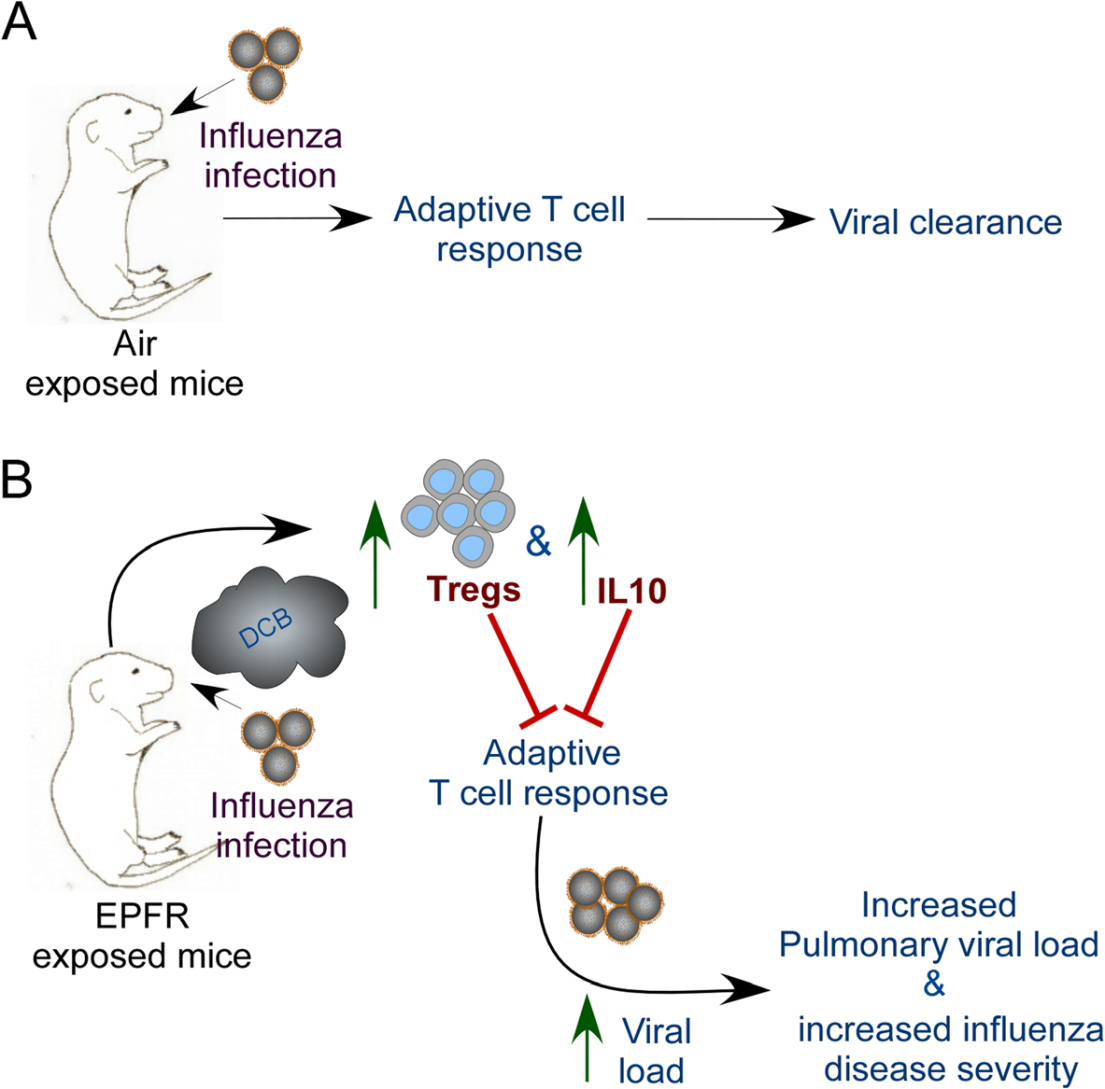
Role of Tregs and IL10 in EPFR-induced exacerbation of influenza disease severity in infected neonatal mice. **a** Influenza infection in Air-exposed neonatal mice induces adequate adaptive T cell response leading to viral clearance. **b** Exposure to EPFRs induces Tregs and IL10 resulting in suppressed adaptive T cell responses leading to enhanced influenza disease severity in infected neonatal mice

## Discussion

Exposure to PM from combustion sources has been shown to affect pulmonary immunologic homeostasis and exacerbate respiratory health effects such as asthma [8, 20, 21]. The association between exposure to elevated levels of PM and enhanced susceptibility to respiratory tract infections in children has been documented. [3, 5, 22–25]. Although studies have indicated this association, very limited information is available on the potential mechanisms and biological rationale associated with these phenomena. Yet, it is important to understand how exposure to elevated levels of PM increase susceptibility to respiratory tract infections. Particle pollutants such as diesel exhaust particles were shown to reduce host defense in adult mice models of influenza infection [26], however studies from our group and other groups have demonstrated that infant responses to infectious agents and PM are markedly different from that of adults [12, 27–29]. These differences could be due to several factors including age and strain of the mice, the composition and dose of PM, the type of the infectious agent, timing of the exposure to PM (before and during vs. after infection), etc. Effects of exposure to PM are particularly detrimental in infants and young children; and children are particularly at risk to respiratory infections compared to adults. Our previous studies with both infectious and non-infectious agents showed that adults and neonates exhibit marked differences in immunological responses [11, 12, 30]. Since infancy is a time of rapid pulmonary and immunological development, insults to the respiratory and immune system during this critical developmental period may result in exaggerated manifestation of disease and/or long-term health effects.

Since infants are particularly susceptible to PM pollution and their immune systems are often characterized as immature, it is important to understand how early life-exposure to EPFR containing PM increases severity of respiratory tract infections such as influenza. Our previous studies demonstrated that early-life exposure to PM alters airway barrier function [9], and our more recent data demonstrates that such exposure in neonates also impairs protective T effector cell responses towards influenza virus infection [10]. Like others, we demonstrated a role for PM-induced oxidative stress [27]; and specifically, we found that particles containing radicals and inducing oxidative stress were necessary for increased viral load and enhanced morbidity and mortality following influenza infection [10]. Apart from realizing that oxidative stress was necessary for this immunosuppressive environment, the mechanism responsible for PM-induced immunosuppression was unclear. Using a neonatal mice model of PM exposure and infection, we have successfully recapitulated the enhanced disease due to respiratory tract viral infection following exposure to elevated levels of PM [10] as reported in several epidemiological studies [3, 5, 22–24]. In the current study, using early-life exposure, we explored the mechanism of EPFR-induced pulmonary immunosuppression and subsequent increase in influenza disease severity.

Here, we demonstrate that Tregs are responsible for creating and maintaining the immunosuppressive environment in the lungs of EPFR-exposed mice. In fact, depletion of Tregs in EPFR-exposed neonatal mice resulted in increased protective T cell responses (i.e. Tc1 and Th1) following influenza infection. Further, adoptive transfer of Tregs from EPFR-exposed neonates to air-exposed neonates suppressed protective T cell responses toward influenza in the recipient air-exposed neonatal mice. We also observed an increase in IL10 in the lungs of EPFR-exposed neonates. Interestingly, in EPFR-exposed neonates IL10 expression was bimodal (i.e., increasing immediately after exposure to DCB, returning to baseline and then peaking at 6 dpi). Administration of rIL10 to air-exposed mice prior to influenza infection recapitulated many of the effects observed in EPFR-exposed mice infected with influenza including increased pulmonary viral load and decreased weight gain. Mice deficient in IL10 exhibited decreased viral load and morbidity compared to WT neonates exposed to EPFRs and infected with influenza virus. Interestingly, Treg and IL10 induction occurred in the absence of influenza infection indicating that early life exposure to EPFRs activates a non-specific immunosuppressive environment in the lungs. Thus, immunosuppression of adaptive T cell responses against influenza appears to be a “bystander” effect that allows for enhanced severity of respiratory tract viral infections.

In addition to Tregs, we observed a significant increase in IL10 expression in the total lung homogenates of neonatal mice exposed to EPFRs. IL10 is an immunoregulatory cytokine produced by a wide range of immune cell types including Tregs and plays a role in regulating the growth and differentiation of various immune cells [31] and in maintaining immune homeostasis by regulating the adaptive immune responses [32, 33]. It inhibits the activity of Th1 cells, NK cells, and macrophages - all of which are required for pathogen clearance during infection [34]. Our data demonstrated that IL10 is sufficient to exacerbate the morbidity and pulmonary viral load associated with influenza infection (Fig. 4b, c, and d, respectively). While influenza infection alone is known to induce increased expression of IL10 at the time of peak adaptive T cell response [35], we also observed an increase in IL10 levels in the lungs of EPFR exposed neonates at 1 dpe (Fig. 1b), prior to influenza infection. Although beyond the scope of this manuscript, early IL10 is most likely produced by monocytes and may play a role in the expansion of inducible Tregs [36].

Our results demonstrating increases in Tregs and IL10 in EPFR-exposed mice are not dissimilar to results from patients infected with the 2009 pandemic influenza A H1N1 virus (pH1N1). Even though these studies looked at different compartments (i.e. peripheral blood versus pulmonary), they also observed an increase in the frequency of Tregs in infected patients that correlated with decreased frequencies of CD3^+^, CD4^+^ and CD8^+^ T cells [37]. And in a separate study, elevated levels of IL10 in sera were positively correlated to severity of disease in patients infected with pH1N1 [38]. It is interesting to postulate that severe lung injury resulting from EPFR exposure in neonatal mice or following pH1N1 infection is a common mechanism responsible for the induction of Tregs and reduction of protective T cells responses and we are currently exploring these possibilities.

Finally, we have previously shown very different immunological responses to similar acute EPFR exposure protocols in adult mice. In fact, Th17 mediated immune responses, which was accompanied by significant pulmonary neutrophilia, predominated in the lungs of adult mice exposed to EPFRs [21]. The reasons for such differences are currently unclear, but they could be a result of differences in maturation status of the immune and/or pulmonary systems.

## Conclusions

Collectively, our studies for the first time demonstrate a novel mechanism by which early-life exposure to EPFR-containing PM induces a non-specific immunosuppressive environment mediated by Tregs and its effector cytokine, IL10. Our data provide insight into how exposure to PM during critical developmental stages of early life plays a role in increased disease severity following LRTI (Fig. 7). These findings validate previous epidemiological and experimental data demonstrating an association between PM exposure and enhanced respiratory tract disease severity. Importantly, these data demonstrate a direct role for EPFR-induced Tregs and IL10 in enhancing influenza morbidity and mortality. The fact that similar responses (increased Tregs and decreased protective T cells) are observed in severe cases of pH1N1 highlights the deleterious effects of early-life exposure to EPFRs.

## Additional files

Additional file 2: Depletion of Tregs in neonatal mice. (A) Timeline for DCB exposure and administration of LEAF purified monoclonal anti-CD25 antibody (PC61 clone). (B), (C) Anti-CD25 antibody administration significantly decreased pulmonary Tregs at 4 and 10 dpe, respectively. **p* < 0.01 vs DCB/Isotype ctrl. Means ± SEM are plotted. Student’s *t*-test. (TIF 232 kb)
Additional file 3: Effect of Treg depletion on IL10 levels in the lungs of EPFR exposed and influenza infected neonatal mice. IL10 levels in the lungs of Treg depleted and EPFR exposed neonatal mice at 6 dpi after infection with influenza virus (DCB/PC61/Flu) in comparison to Air/Flu, DCB/Flu, and EPFR exposed neonatal mice treated with rat IgG isotype control and infected with influenza virus (DCB/Isotype/flu). Data are plotted as means ± SEM, **p* < 0.05. One-way ANOVA with Holm-Sidak’s multiple comparisons test. (DOCX 12 kb)
Additional file 4: Isolation of Tregs and determination of purity using flow cytometry. Tregs were isolated from DCB exposed neonates at 10 dpe. Lungs were isolated from DCB exposed neonates at 10 dpe and single cell suspension was purified for Tregs by a two-step protocol. Isolated single cells were enriched for CD4^+^ T cells followed by isolation of CD25^+^ cells. After the final isolation, single cells were analyzed for efficiency of Treg isolation using flow cytometry. Panels A and B represent the CD25 positive fraction. Panels C and D represent the CD25 negative fraction. Percentage of CD4^+^ cells (CD4^hi^ and CD4^mid^) (Panels A and C) with distribution of CD4^hi^ CD25 and CD4^mid^ CD25 cells (Panels B and D) represented in dot plots. The isolated CD25 positive fraction consisted of ~89% CD4^+^ cells with ~69% CD4^mid^ CD25^+^ cells and ~14% CD4^hi^ CD25^+^ cells, whereas 0.01% CD4^mid^ CD25^+^ cells and ~1% CD4^hi^ CD25^+^ cells were found in the CD25 negative fraction. (TIF 175 kb)
Additional file 1: Time-course of TNFα levels after exposure to EPFRs and influenza infection. Lungs were isolated on 1 and 4 dpe and on 1 and 6 dpi and whole lung homogenates were analyzed for TNFα levels using ELISA. TNFα was found to be below the limit of detection (1.5 pg/ml) in Air and DCB exposed lungs at 1 and 4 dpe and in Air/Flu and DCB/Flu mice at 1 dpi. Detectable levels of TNFα were found only in 3 of the DCB/Flu (*n* = 6) mice at 6 dpi. (TIF 18 kb)

## Abbreviations

ANOVA: Analysis of variance
COPD: Chronic obstructive pulmonary disease
DCB: EPFR containing PM
DPBS: Dulbecco’s phosphate-buffered saline
dpe: Days post exposure
dpi: Day post-infection
EPFR: Environmentally persistent free radicals
HBSS: Hank’s balanced salt solution
i.n.: Intranasal
LRTI: Lower respiratory tract infection
rIL10: Recombinant IL10
SEM: Standard error of means
Tc1: CD8+ T cell expressing IFNγ
Th1: CD4+ T cell expressing IFNγ
T-PER: Tissue protein extract reagent
Treg: Regulatory T cells
Tregs: Regulatory T cells

## References

[1] Gehring U, Wijga AH, Hoek G, Bellander T, Berdel D, Bruske I, Fuertes E, Gruzieva O, Heinrich J, Hoffmann B, de Jongste JC, Klumper C, Koppelman GH, Korek M, Kramer U, Maier D, Melen E, Pershagen G, Postma DS, Standl M, von Berg A, Anto JM, Bousquet J, Keil T, Smit HA, Brunekreef B (2015). Exposure to air pollution and development of asthma and rhinoconjunctivitis throughout childhood and adolescence: a population-based birth cohort study. Lancet Respir Med.

[2] Ling SH, van Eeden SF (2009). Particulate matter air pollution exposure: role in the development and exacerbation of chronic obstructive pulmonary disease. Int J Chron Obstruct Pulmon Dis.

[3] Smith KR, Samet JM, Romieu I, Bruce N (2000). Indoor air pollution in developing countries and acute lower respiratory infections in children. Thorax.

[4] Lin M, Stieb DM, Chen Y (2005). Coarse particulate matter and hospitalization for respiratory infections in children younger than 15 years in Toronto: a case-crossover analysis. Pediatrics.

[5] Gurley ES, Homaira N, Salje H, Ram PK, Haque R, Petri W, Bresee J, Moss WJ, Breysse P, Luby SP, Azziz-Baumgartner E (2013). Indoor exposure to particulate matter and the incidence of acute lower respiratory infections among children: a birth cohort study in urban Bangladesh. Indoor Air.

[6] Dellinger B, Pryor WA, Cueto R, Squadrito GL, Hegde V, Deutsch WA (2001). Role of free radicals in the toxicity of airborne fine particulate matter. Chem Res Toxicol.

[7] Saravia J, Lee GI, Lomnicki S, Dellinger B, Cormier SA (2013). Particulate matter containing environmentally persistent free radicals and adverse infant respiratory health effects: a review. J Biochem Mol Toxicol.

[8] Balakrishna S, Saravia J, Thevenot P, Ahlert T, Lominiki S, Dellinger B, Cormier SA (2011). Environmentally persistent free radicals induce airway hyperresponsiveness in neonatal rat lungs. Part Fibre Toxicol.

[9] Thevenot PT, Saravia J, Jin N, Giaimo JD, Chustz RE, Mahne S, Kelley MA, Hebert VY, Dellinger B, Dugas TR, Demayo FJ, Cormier SA (2013). Radical-containing ultrafine particulate matter initiates epithelial-to-mesenchymal transitions in airway epithelial cells. Am J Respir Cell Mol Biol.

[10] Lee GI, Saravia J, You D, Shrestha B, Jaligama S, Hebert VY, Dugas TR, Cormier SA (2014). Exposure to combustion generated environmentally persistent free radicals enhances severity of influenza virus infection. Part Fibre Toxicol.

[11] Saravia J, You D, Thevenot P, Lee GI, Shrestha B, Lomnicki S, Cormier SA (2014). Early-life exposure to combustion-derived particulate matter causes pulmonary immunosuppression. Mucosal Immunol.

[12] You D, Ripple M, Balakrishna S, Troxclair D, Sandquist D, Ding L, Ahlert TA, Cormier SA (2008). Inchoate CD8+ T cell responses in neonatal mice permit influenza-induced persistent pulmonary dysfunction. J Immunol.

[13] Lomnicki S, Truong H, Vejerano E, Dellinger B (2008). Copper oxide-based model of persistent free radical formation on combustion-derived particulate matter. Environ Sci Technol.

[14] Balakrishna S, Lomnicki S, McAvey KM, Cole RB, Dellinger B, Cormier SA (2009). Environmentally persistent free radicals amplify ultrafine particle mediated cellular oxidative stress and cytotoxicity. Part Fibre Toxicol.

[15] Fahmy B, Ding L, You D, Lomnicki S, Dellinger B, Cormier SA (2010). In vitro and in vivo assessment of pulmonary risk associated with exposure to combustion generated fine particles. Environ Toxicol Pharmacol.

[16] Kannan R, Guo P, Przekwas A: Particle transport in the human respiratory tract: formulation of a nodal inverse distance weighted Eulerian–Lagrangian transport and implementation of the Wind-Kessel algorithm for an oral delivery. Int J Numer Method Biomed Eng. 2016;32. 10.1002/cnm.2746 26317686

[17] Lai YL, Chou H (2000). Respiratory mechanics and maximal expiratory flow in the anesthetized mouse. J Appl Physiol (1985).

[18] Reed LJ, Muench H (1938). A simple method of estimating fifty per cent endpoints. Am J Epidemiol.

[19] MG O. Preparing suspensions of single cells. In Flow Cytometry: A practical approach. 3rd edition. Chippenham: Oxford University Press; 2000:35–46.

[20] Jaligama S, Chen Z, Saravia J, Yadav N, Lomnicki SM, Dugas TR, Cormier SA (2015). Exposure to deepwater horizon crude Oil burnoff particulate matter induces pulmonary inflammation and alters adaptive immune response. Environ Sci Technol.

[21] Wang P, Thevenot P, Saravia J, Ahlert T, Cormier SA (2011). Radical-containing particles activate dendritic cells and enhance Th17 inflammation in a mouse model of asthma. Am J Respir Cell Mol Biol.

[22] Darrow LA, Klein M, Flanders WD, Mulholland JA, Tolbert PE, Strickland MJ (2014). Air pollution and acute respiratory infections among children 0–4 years of age: an 18-year time-series study. Am J Epidemiol.

[23] Wong CM, Thach TQ, Chau PY, Chan EK, Chung RY, Ou CQ, Yang L, Peiris JS, Thomas GN, Lam TH, Wong TW, Hedley AJ, Committee HEIHR: Part 4. Interaction between air pollution and respiratory viruses: time-series study of daily mortality and hospital admissions in Hong Kong. Res Rep Health Eff Inst. 2010;283–362. 21446214

[24] Fukuda K, Hider PN, Epton MJ, Jennings LC, Kingham SP (2011). Including viral infection data supports an association between particulate pollution and respiratory admissions. Aust N Z J Public Health.

[25] Braga AL, Saldiva PH, Pereira LA, Menezes JJ, Conceicao GM, Lin CA, Zanobetti A, Schwartz J, Dockery DW (2001). Health effects of air pollution exposure on children and adolescents in Sao Paulo, Brazil. Pediatr Pulmonol.

[26] Gowdy KM, Krantz QT, King C, Boykin E, Jaspers I, Linak WP, Gilmour MI (2010). Role of oxidative stress on diesel-enhanced influenza infection in mice. Part Fibre Toxicol.

[27] Chan JK, Charrier JG, Kodani SD, Vogel CF, Kado SY, Anderson DS, Anastasio C, Van Winkle LS (2013). Combustion-derived flame generated ultrafine soot generates reactive oxygen species and activates Nrf2 antioxidants differently in neonatal and adult rat lungs. Part Fibre Toxicol.

[28] You D, Becnel D, Wang K, Ripple M, Daly M, Cormier SA (2006). Exposure of neonates to respiratory syncytial virus is critical in determining subsequent airway response in adults. Respir Res.

[29] You D, Marr N, Saravia J, Shrestha B, Lee GI, Turvey SE, Brombacher F, Herbert DR, Cormier SA (2013). IL-4Ralpha on CD4+ T cells plays a pathogenic role in respiratory syncytial virus reinfection in mice infected initially as neonates. J Leukoc Biol.

[30] Saravia J, You D, Shrestha B, Jaligama S, Siefker D, Lee GI, Harding JN, Jones TL, Rovnaghi C, Bagga B, DeVincenzo JP, Cormier SA (2015). Respiratory syncytial virus disease is mediated by Age-variable IL-33. PLoS Pathog.

[31] Moore KW, de Waal MR, Coffman RL, O'Garra A (2001). Interleukin-10 and the interleukin-10 receptor. Annu Rev Immunol.

[32] Ng TH, Britton GJ, Hill EV, Verhagen J, Burton BR, Wraith DC (2013). Regulation of adaptive immunity; the role of interleukin-10. Front Immunol.

[33] Chaudhry A, Samstein RM, Treuting P, Liang Y, Pils MC, Heinrich JM, Jack RS, Wunderlich FT, Bruning JC, Muller W, Rudensky AY (2011). Interleukin-10 signaling in regulatory T cells is required for suppression of Th17 cell-mediated inflammation. Immunity.

[34] Couper KN, Blount DG, Riley EM (2008). IL-10: the master regulator of immunity to infection. J Immunol.

[35] Sun K, Torres L, Metzger DW (2010). A detrimental effect of interleukin-10 on protective pulmonary humoral immunity during primary influenza A virus infection. J Virol.

[36] Hsu P, Santner-Nanan B, Hu M, Skarratt K, Lee CH, Stormon M, Wong M, Fuller SJ, Nanan R (2015). IL-10 potentiates differentiation of human induced regulatory T cells via STAT3 and Foxo1. J Immunol.

[37] Huang Y, Zhu W, Zeng X, Li S, Li X, Lu C (2013). Innate and adaptive immune responses in patients with pandemic influenza A(H1N1)pdm09. Arch Virol.

[38] Yu X, Zhang X, Zhao B, Wang J, Zhu Z, Teng Z, Shao J, Shen J, Gao Y, Yuan Z, Wu F (2011). Intensive cytokine induction in pandemic H1N1 influenza virus infection accompanied by robust production of IL-10 and IL-6. PLoS One.

