# Supplementary figures and images for: Regulatory T cells and IL10 suppress pulmonary host defense during early-life exposure to radical containing combustion derived ultrafine particulate matter
Source: Respir Res. 2017 Jan 13;18:15. doi: 10.1186/s12931-016-0487-4 (PMC5237352; doi:10.1186/s12931-016-0487-4)

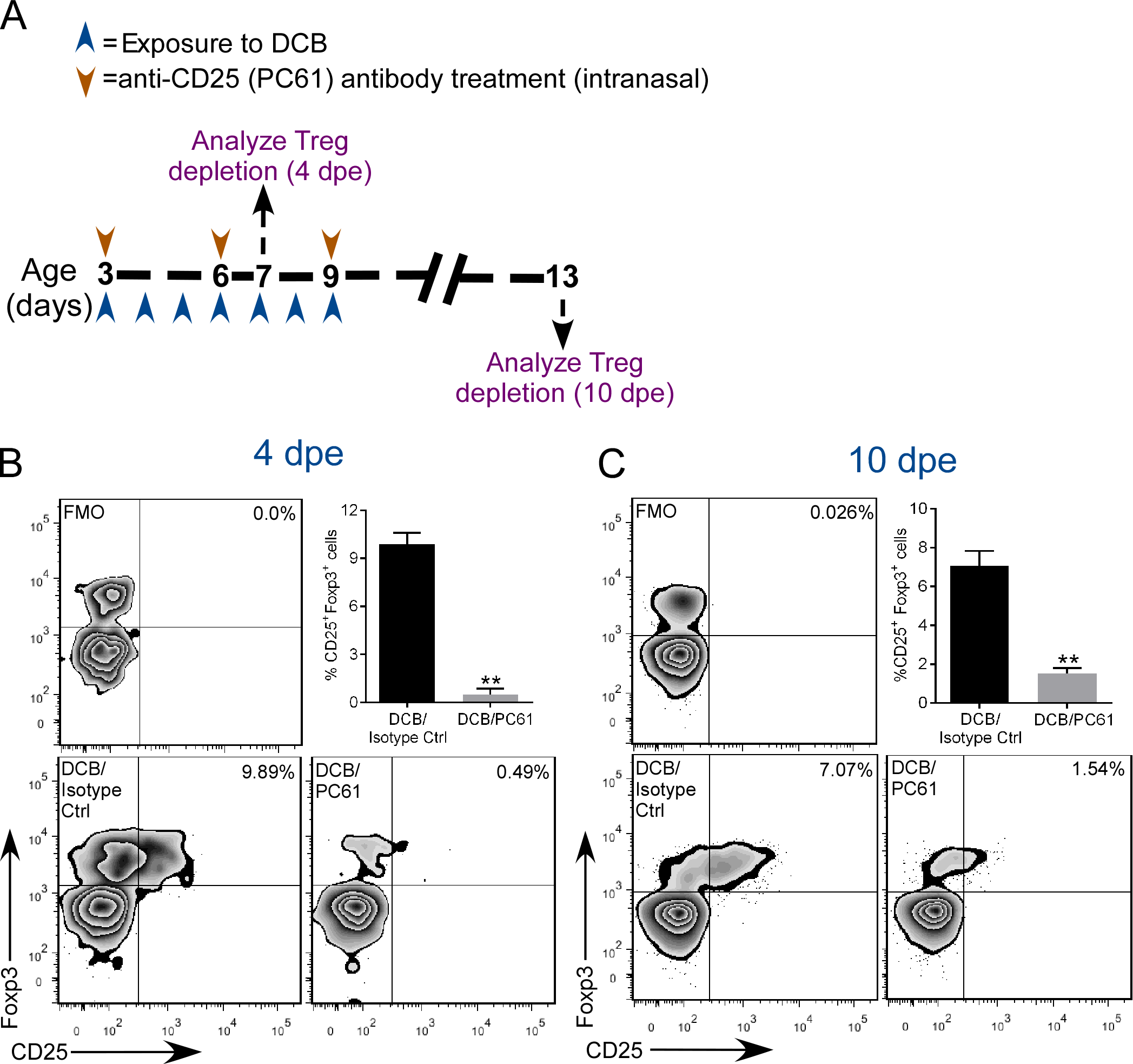

Supplement: Additional file 2: — Depletion of Tregs in neonatal mice. (A) Timeline for DCB exposure and administration of LEAF purified monoclonal anti-CD25 antibody (PC61 clone). (B), (C) Anti-CD25 antibody administration significantly decreased pulmonary Tregs at 4 and 10 dpe, respectively. *p < 0.01 vs DCB/Isotype ctrl. Means ± SEM are plotted. Student’s t-test. (TIF 232 kb) [file 12931_2016_487_MOESM2_ESM.tif]

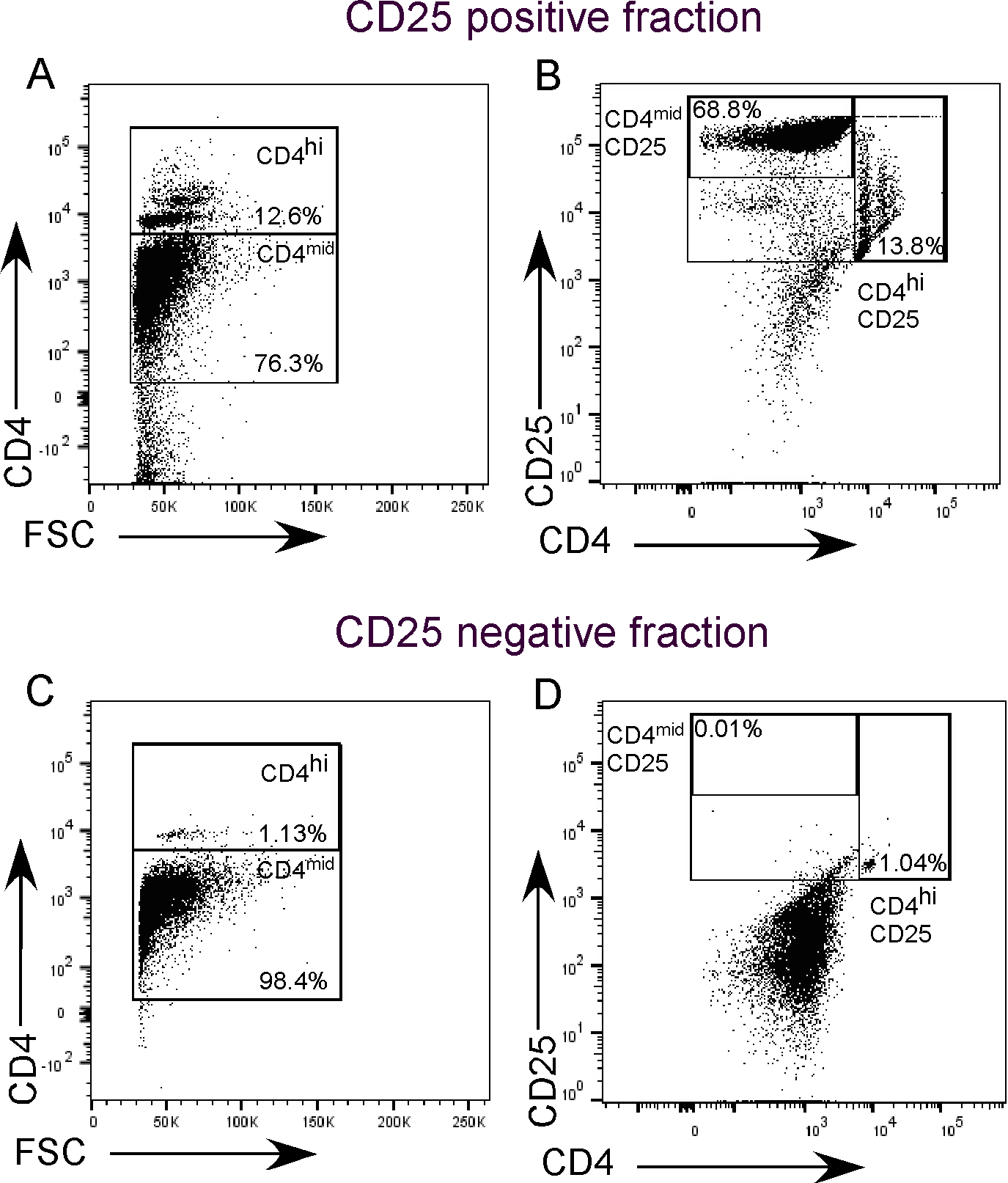

Supplement: Additional file 4: — Isolation of Tregs and determination of purity using flow cytometry. Tregs were isolated from DCB exposed neonates at 10 dpe. Lungs were isolated from DCB exposed neonates at 10 dpe and single cell suspension was purified for Tregs by a two-step protocol. Isolated single cells were enriched for CD4+ T cells followed by isolation of CD25+ cells. After the final isolation, single cells were analyzed for efficiency of Treg isolation using flow cytometry. Panels A and B represent the CD25 positive fraction. Panels C and D represent the CD25 negative fraction. Percentage of CD4+ cells (CD4hi and CD4mid) (Panels A and C) with distribution of CD4hi CD25 and CD4mid CD25 cells (Panels B and D) represented in dot plots. The isolated CD25 positive fraction consisted of ~89% CD4+ cells with ~69% CD4mid CD25+ cells and ~14% CD4hi CD25+ cells, whereas 0.01% CD4mid CD25+ cells and ~1% CD4hi CD25+ cells were found in the CD25 negative fraction. (TIF 175 kb) [file 12931_2016_487_MOESM4_ESM.tif]
