# Supplementary material for: Regulatory T cells and IL10 suppress pulmonary host defense during early-life exposure to radical containing combustion derived ultrafine particulate matter
Source: Respir Res. 2017 Jan 13;18:15. doi: 10.1186/s12931-016-0487-4 (PMC5237352; doi:10.1186/s12931-016-0487-4)
Supplement: Additional file 3: — Effect of Treg depletion on IL10 levels in the lungs of EPFR exposed and influenza infected neonatal mice. IL10 levels in the lungs of Treg depleted and EPFR exposed neonatal mice at 6 dpi after infection with influenza virus (DCB/PC61/Flu) in comparison to Air/Flu, DCB/Flu, and EPFR exposed neonatal mice treated with rat IgG isotype control and infected with influenza virus (DCB/Isotype/flu). Data are plotted as means ± SEM, *p < 0.05. One-way ANOVA with Holm-Sidak’s multiple comparisons test. (DOCX 12 kb) [file 12931_2016_487_MOESM3_ESM.docx]

**Effect of Treg depletion on IL10 levels in the lungs of EPFR exposed and influenza infected neonatal mice.**

| **Group ID** | **IL10 (pg/mg)**  **Mean ± SEM** |
| --- | --- |
| **Air/Flu** | 11.43 ± 0.38 |
| **DCB/Flu** | 14.69 ± 1.52 |
| **DCB/Isotype/Flu** | 11.85 ± 0.54 |
| **DCB/PC61/Flu** | 8.08 ± 0.69^*#^ |

IL10 levels in the lungs of Treg depleted and EPFR exposed neonatal mice at 6 dpi after infection with influenza virus (DCB/PC61/Flu) in comparison to Air/Flu, DCB/Flu, and EPFR exposed neonatal mice treated with rat IgG isotype control and infected with influenza virus (DCB/Isotype/flu). Data are plotted as means ± SEM, *p<0.05 DCB/Flu vs DCB/PC61/Flu, ^#^ p<0.05 DCB/Isotype/Flu vs DCB/PC61/Flu. One-way ANOVA with Holm-Sidak’s multiple comparisons test.
